# TiO_2_/HA and Titanate/HA Double-Layer Coatings on Ti6Al4V Surface and Their Influence on In Vitro Cell Growth and Osteogenic Potential

**DOI:** 10.3390/jfb13040271

**Published:** 2022-12-01

**Authors:** Michalina Ehlert, Aleksandra Radtke, Natalia Forbot, Tomasz Jędrzejewski, Katarzyna Roszek, Patrycja Golińska, Grzegorz Trykowski, Piotr Piszczek

**Affiliations:** 1 Faculty of Chemistry, Nicolaus Copernicus University in Toruń, Gagarina 7, 87-100 Toruń, Poland; 2 Nano-implant Ltd., Gagarina 7/47, 87-100 Toruń, Poland; 3 Faculty of Biological and Veterinary Sciences, Nicolaus Copernicus University in Toruń, Lwowska 1, 87-100 Toruń, Poland

**Keywords:** hydroxyapatite, titanate nanolayers, titanium dioxide, cathodic electrodeposition, biointegration, antimicrobial activity, adipose-derived mesenchymal stem cells

## Abstract

Hydroxyapatite (HA) layers are appropriate biomaterials for use in the modification of the surface of implants produced inter alia from a Ti6Al4V alloy. The issue that must be solved is to provide implants with appropriate biointegration properties, enabling the permanent link between them and bone tissues, which is not so easy with the HA layer. Our proposition is the use of the intermediate layer ((IL) = TiO_2_, and titanate layers) to successfully link the HA coating to a metal substrate (Ti6Al4V). The morphology, structure, and chemical composition of Ti6Al4V/IL/HA systems were characterized by scanning electron microscopy (SEM), X-ray diffraction (XRD), and energy-dispersive X-ray spectrometry (EDS). We evaluated the apatite-forming ability on the surface of the layer in simulated body fluid. We investigated the effects of the obtained systems on the viability and growth of human MG-63 osteoblast-like cells, mouse L929 fibroblasts, and adipose-derived human mesenchymal stem cells (ADSCs) in vitro, as well as on their osteogenic properties. Based on the obtained results, we can conclude that both investigated systems reflect the physiological environment of bone tissue and create a biocompatible surface supporting cell growth. However, the nanoporous TiO_2_ intermediate layer with osteogenesis-supportive activity seems most promising for the practical application of Ti6Al4V/TiO_2_/HA as a system of bone tissue regeneration.

## 1. Introduction

Tissue engineering aims to replace, restore, improve or maintain the function of tissues and organs using implants containing the patient’s cells embedded in a special biomaterial that acts as a cell scaffold [1,2,3,4]. The composition, architecture, and possibility of resorption are factors that determine the biomatrix’s biocompatibility. Biomaterials are used for several different types of implants, such as surgical, orthopedic, dental, craniofacial, and arthroplasty applications. The implant function in the human body is a key feature for the requirements to be achieved by the materials used in their construction [4,5,6,7,8,9,10,11]. However, they must also possess the appropriate mechanical strength and porosity to allow cell adhesion [5,12,13,14]. The material’s biocompatibility is a significant property that should be considered during their choice for implant construction, which will prevent its rejection by the body after implantation. A major risk is the loosening or fracture of the implant (shielding effect), which can cause painful inflammation and infection of the surrounding tissues [15,16,17]. In addition, it is important to ensure that its production process is reproducible for different batches of devices and that the material does not undergo changes in shape or properties during its sterilization process [15,16,17,18,19,20,21]. For this reason, various innovative technologies (e.g., plasma treatment, low-intensity pulsed ultrasound, magnetic field stimulation, anodization, chemical treatment) [22,23,24,25,26,27,28,29], modifications (e.g., incorporation/deposition of various metal ions) [28,30,31,32,33,34] for the fabrication of biomaterials are being applied to prevent unwanted complications.

The most used material for orthopedic and dental implants is the titanium alloy Ti6Al4V. This is due to its superior corrosion resistance, high fracture resistance, low density, and biocompatibility. Unfortunately, the main problem is its inability to integrate with bone [23,35,36,37,38,39]. Moreover, titanium and titanium alloy show unsatisfactory mechanical properties. The Ti6Al4V alloy (~210 GPa) has a significantly higher elastic modulus compared to human cortical bone tissue (Young’s modulus 10–30 GPa, hardness 0.3–0.7 GPa) and a higher hardness [40,41,42]. Therefore, numerous modifications of its surface are being carried out to make it bioactive. An analysis of previous reports revealed that the fabrication of nanoporous, nanotube, nanosponge-like, and nanofibrous TiO_2_ and titanate coatings on the surface of the Ti6Al4V alloy significantly improves its biointegration properties [29,43,44,45,46,47,48,49,50]. The production of nanocoatings with different morphologies on the surface of the titanium alloy definitely improves its mechanical properties, but it still has not been developed as a biomaterial with very similar mechanical parameters to bone [38,51]. The attractive biomaterial for scaffolding in tissue engineering is hydroxyapatite (Ca_10_(PO_4_)_6_(OH)_2_, HA). Its composition and structure are very similar to the inorganic component of the bone matrix. The main limitation in the use of hydroxyapatite layers is their poor adhesion to metal substrates [52,53,54,55]. Over the past several years, a series of studies have been conducted in an effort to improve the evaluation of implant materials with a hydroxyapatite layer [37,52,53,54,55,56,57,58,59].

It is important to evaluate fabricated systems for their ability to promote bone repair and regeneration. The production of a porous scaffold facilitates increased cell migration and the diffusion of signaling molecules as well as nutrients [60,61,62,63]. The biomaterial should exhibit the potential to mimic the native extracellular matrix (ECM) and support many tissues’ morphogenesis. In regenerative medicine and tissue engineering, there is a growing focus on using mesenchymal stem cells (MSCs) due to their ability to self-renew, proliferate and differentiate toward bone-forming cells [60,61,63,64,65]. Advances in stem cell knowledge have opened new possibilities for obtaining unlimited sources of cells. MSCs are generally isolated from bone marrow. However, they can also be isolated from, e.g., adipose tissue, umbilical cord, muscles, bone, synovium, blood, cartilage or tendon [63,64,66,67,68]. Adipose-derived human mesenchymal stem cells (ADSCs) are increasingly being used in cell therapy development, especially due to their angiogenic potential. Their undoubted advantages compared to stem cells isolated from bone marrow are their easy availability in the body and their non-invasive methods of collection [69,70,71]. The formation of a biocompatible scaffold, alone or in combination with stem cells, is a promising tool to improve the regeneration and repair of bone tissue [29,43,44,66,72].

In research carried out so far, we have focused on developing a manufacturing method for composite systems consisting of a Ti6Al4V alloy/intermediate layer ((IL); TiO_2_ or titanate)/hydroxyapatite layer (HA). The obtained results revealed that the use of oxide intermediate layers linked the Ti6Al4V alloy substrate and the hydroxyapatite surface layer, and significantly improved the mechanical properties of the system. The mechanical parameters (i.e., hardness, Young’s modulus) obtained for the hydroxyapatite layer were similar to cortical bone. Furthermore, the adhesion strength between the titanium alloy substrate and the hydroxyapatite layer was significantly increased by the use of intermediate coatings [30]. Nevertheless, the produced materials with a hydroxyapatite layer should also show high biocompatibility and biointegration properties.

For this reason, in the presented work, we focused on comparing the biological activity of the produced systems with the hydroxyapatite layer, which showed the best mechanical properties. We conducted in vitro studies of the biomaterials mentioned above as Ti6Al4V/IL/HA systems, investigating their effects on the survival and proliferation of cultures of human MG-63 osteoblast-like cells, mouse L929 fibroblast and adipose-derived human mesenchymal stem cells (ADSCs) seeded on their surface. We also estimated the potential antimicrobial activity of the produced systems. These studies allowed for the development of a system with a hydroxyapatite layer, in which a balance between mechanical and biological properties was achieved.

## 2. Materials and Methods

### 2.1. Synthesis of TiO_2_/HA and Titanate/HA Double-Layer Coating

Ti6Al4V/IL/HA composite materials were selected for biological studies, which exhibit physicochemical as well as mechanical properties desirable for biomedical applications. The following intermediate layers (ILs) were selected for biological tests as follows: nanoporous TiO_2_ (T5), nanofibrous TiO_2_ (TNF6C), and titanate (T-S), whose syntheses have been described in detail in our previous reports [29,43,44].

**Figure 1:**
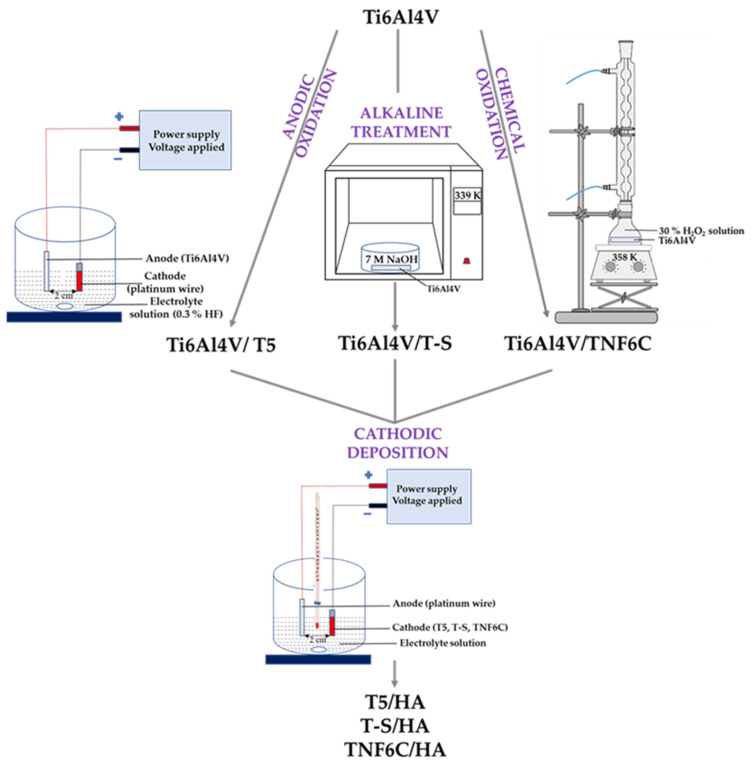
Scheme to produce systems with a hydroxyapatite layer.

The overall scheme for producing the Ti6Al4V/IL/HA composites is presented in Figure 1. The intermediate layers on the surface of the Ti6Al4V substrates were synthesized in the first stage. In all our experiments, 0.20 mm-thick Ti6Al4V alloy foil was used (marked as T, grade 5, 99.7% purity; Strem Chemicals, Inc., Bischheim, France). The electrochemical method of anodic oxidation in 0.3% hydrofluoric acid solution (t = 20 min, U = 5 V) was used to produce TiO_2_ nanoporous coatings (T5). As a result of etching in a ca. 5.8 M hydrochloric acid solution and chemical oxidation in 30% hydrogen peroxides solution (t = 6 h, T = 85 °C under a reflux condenser), TiO_2_ nanofiber coatings (TNF6C) were obtained. Alkali-sodium treatment of the titanium alloy in 7 M sodium hydroxide solution (t = 48 h, T = 65 °C) led to titanate coatings (T-S).

The hydroxyapatite coating was deposited onto the Ti6Al4V/IL system in the next stage. The synthesis, structural and morphological characterization, as well as the physicochemical and mechanical properties of the TiO_2_/HA and titanate/HA double layers have been previously reported [30]. T5, T-S, and TNF6C materials were cut into pieces 6 mm × 100 mm and 10 × 60 mm and 0.2 mm thick. A hydroxyapatite (HA) coating was deposited onto these biomaterials using cathodic electrochemical deposition (t = 60 min, T = 65 °C, I = 2.5 mA for T5 and T-S samples and I = 3.5 mA for TNF6C samples; pH of the electrolytes = 4.5). The electrolyte consisted of components dissolved in distilled water: Ca(NO_3_)_2_ · 4 H_2_O (0.042 M), NH_4_H_2_PO_4_ (0.025 M) and EDTA-2Na (1.5 × 10^−^^3^). Then, the samples were immersed in 0.1 M NaOH solution (t = 2 h, T = 65 °C) and finally sintered (t = 2 h, T = 250 °C).

All specimens prepared for bioassay were sterilized (t = 20 min, T = 123 °C, p = 120 kPa) with an IS YESON YS-18L autoclave (Yeson, Ningbo, China).

### 2.2. SEM and Element Analysis

Scanning electron microscopy (SEM) studies were carried out using two microscopes: (1) high-resolution with field emission electron source (HR-SEM, Quanta 3D FEG, FEI Company, Brno, Czech Republic); (2) tungsten cathode microscope (SEM, EVO 15, Carl Zeiss Microscopy, Oberkochen, Germany) coupled to energy-dispersive X-ray spectrometer (EDS, SmartEDS, Carl Zeiss Microscopy, Oberkochen, Germany), which was used to analyze the elements contained in all the synthesized double layers. Measurements on both microscopes were made in the variable pressure mode (VP, with a pressure of 50 Pa in the chamber). X-ray diffraction analyses were carried out in the PANalytical X’Pert Pro model diffractometer (Malvern PANalytical B.V., Almelo, The Netherlands) with Cu-K alfa radiation and grazing incidence angle mode (GIXRD; the incidence angle was equal to 1 degree).

### 2.3. Apatite-Forming Ability

In accordance with ISO/FDIS 23317:2007(E) and Kokubo’s formulation, we evaluated the apatite-forming ability on the surface of the layers in simulated body fluid (SBF) [73,74]. By immersing the samples in SBF solution, we wanted to (a) assess the stability of the HA layers linked through an intermediate layer (IL) to the Ti6Al4V substrate, as well as to (b) verify whether the produced HA layer promotes further apatite growth in a solution of similar composition to the human body fluids. These studies were carried out for the T5/HA, T-S/HA and TNF6C/HA systems. Immersion in SBF solution was carried out according to the procedure we described earlier at a constant temperature of 36.5 °C for 7, 14, 21, and 28 days, and each sample was kept in a vertical position inside polypropylene tubes [43]. The percentage weight gain observed after removing and drying the samples from the SBF solution was calculated.

### 2.4. Cell Culture

L929 mouse fibroblast cells were obtained from the American Type Culture Collection (Manassas, VA, USA) and cultivated in RPMI 1640 medium supplemented with 10% fetal bovine serum (FBS) and antibiotics (penicillin and streptomycin). Human osteoblast-like MG-63 cells were purchased from the European Collection of Cell Cultures (Salisbury, UK) and cultured in EMEM medium supplemented with L-glutamine sodium pyruvate, non-essential amino acid, 10% FBS, and antibiotics. Adipose-derived human mesenchymal stem cells (ADSCs) were purchased from PromoCell (Heidelberg, Germany) and cultivated in Mesenchymal Stem Cell Growth Medium^®^ containing 10% Supplement Mix^®^ and antibiotics. All cell lines were cultured at 37 °C under a humidified atmosphere of 5% CO_2_. After reaching approximately 70% cell density, the cells were trypsinized using trypsin/EDTA solution at a concentration of 0.25% for L929 and MG-63 cells or 0.04% for ADSCs, respectively. The reagents used for the L929 and MG-63 cell cultures were obtained from Merck KGaA (Darmstadt, Germany), whereas those for ADSCs were purchased from PromoCell.

### 2.5. Cell Proliferation Assay

The MTT (3-(4,5-dimethylthiazolyl)-2,5-diphenyl-tetrazolium bromide; Merck KGaA, Darmstadt, Germany) assays were used to evaluate cell proliferation. Firstly, 1 × 10^4^ L929 fibroblasts, 1 × 10^4^ MG-63 cells and 3 × 10^4^ ADSCs were seeded in a 10 µL drop onto the sterile scaffolds placed in a 24-well plate and were left for 4 h to adhere. Then, 500 µL of suitable culture medium was added and the cells were cultured at 37 °C for 1, 5 and 7 days. Then, the samples were rinsed with phosphate-buffered saline (PBS; Merck KGaA) and 100 µL of MTT solution at a concentration of 0.5 mg/mL was added to each well. After 3 h of incubation at 37 °C, the cells were washed again with PBS and 300 μL of dimethyl sulfoxide (DMSO; 100% *v*/*v*) were added to each well. The formed formazan crystals were dissolved by shaking plates for 10 min. The optical density was measured at 570 nm (with reference wavelength of 630 nm) using Synergy HT Multi-detection reader (BioTek Instruments, Winooski, VT, USA). The blank samples without cells were treated identically to the experimental scaffolds.

### 2.6. Analysis of Cells Using Scanning Electron Microscopy

The analysis of L929, MG-63, ADSC cell morphologies and the levels of proliferation over time on the selected scaffolds (T5/HA and TNF6C/HA) was conducted using scanning electron microscopy (EVO 15). The cells were seeded on the specimens at the same density as in the MTT assays and were cultured for 1 and 5 days. Then, the scaffolds were rinsed with PBS, fixed in 2.5% *v*/*v* glutaraldehyde and dehydrated in a graded series of ethanol concentrations (50%, 75%, 90%, and 100%) for 20 min at each ethanol concentration. Finally, the specimens were dried overnight before the SEM analysis was performed.

### 2.7. Alizarin Red S Staining

Before staining, cells were fixed for 15 min in 10% formalin solution, then residual formaldehyde was removed by washing the wells twice with bi-distilled water. Extracellular calcium deposits were stained through 20 min incubation with 500 µL Alizarin Red solution. Unbound dye residues were rinsed 4 times for 5 min with bi-distilled water. For quantitative analysis, stained calcium deposits were mechanically removed from titanium substrates with a scraper and then bound dye was dissolved in 500 µL 10% acetic acid by shaking for 30 min in 37 °C. The result was measured spectrophotometrically at a wavelength of 405 nm. To avoid a false positive result due to deposition of the dye on the titanium plates coated with hydroxyapatite, control stains were performed on the plates without seeded cells and these values were subtracted from the test samples.

### 2.8. Alkaline Phosphatase (ALP) Activity

The first step in determining the enzyme activity was to perform cell lysates. First, 350 µL of lysis buffer were added to each well, incubated for 10 min at 37 °C, and cells were mechanically disintegrated with a scraper. The obtained lysates were centrifuged (3 min, 3000× *g*) and 300 µL were transferred to the wells to determine the catalytic activity of the enzyme.

The substrate used for the enzymatic reaction was 1 mM p-nitrophenylphosphate (pNPP). Activity was measured by adding 0.3 mL of substrate solution to 0.3 mL of cell lysate. The blank sample—0.3 mL of lysis buffer and 0.3 mL of substrate—was also prepared. All samples were incubated for 1 h at 37 °C and then the reaction was stopped by adding 0.2 mL of 1% NaOH solution. The absorbance of the samples was measured at 405 nm by using Synergy HT Multi-detection reader. The produced p-nitrophenol concentration was calculated using the calibration curve, and ALP activity was normalized to cell number in appropriate samples.

### 2.9. Antimicrobial Activity

Biocidal activity of the selected scaffolds with hydroxyapatite layers were estimated against Gram-positive (Staphylococcus aureus ATCC 25923, Staphylococcus aureus ATCC 6538), Gram-negative (Escherichia coli ATCC 25922, Escherichia coli ATCC 8739) bacteria and Candida albicans ATCC 10231. The scaffolds were placed in the 12-well plates with 1 mL of microbial inoculum (1.0 − 3.3 *×* 10^6^ C.F.U mL^−^^1^) in 1 x phosphate-buffered saline (PBS) without ions (EurX) and incubated for 24 h at 37 °C. Buffer was sterilized by filtration through 0.22 µm filters prior to use. Microbial density was prepared using a densitometer (Biosan, Latvia), diluted accordingly with PBS to the final concentration and estimated by colony counts after the spreading of 100 µL on Triptic Soy Agar (TSA; Becton Dickinson, USA for bacteria) or Sabouraus Dextrose Agar (SDA; Becton-Dickinson for *C. albicans*). The positive control was the inoculum without scaffolds. After incubation, the inoculum was collected from the wells, ten-fold diluted and spread (100 µL) on the appropriate medium in Petri dishes. Plates were incubated for 24h at 37 °C. Colony-forming units were counted on the inoculated plates and compared with the appropriate control plates to estimate the reduction of bacterial or fungal growth.

The antimicrobial activity was determined based on the reduction (R) factor calculated according to the formula R = Ut – At, where Ut is the common logarithm of the number of bacteria in the untreated microbial suspension and At is the common logarithm of the number of bacteria in the treated microbial suspension. R ≥ 2 determines the biocidal activity of the tested sample.

### 2.10. Statistical Analysis

All data are presented as mean ± SEM and were evaluated using a one-way analysis of variance (ANOVA) followed by Tukey’s post-hoc multiple comparisons test. Significance level was set at *p* < 0.05. GraphPad Prism 7.0 software (GraphPad Software Inc., La Jolla, CA, USA) was used to perform statistical analyses.

## 3. Results

### 3.1. Surface Morphology of TiO_2_/HA and Titanate/HA Double-Layer Coating

**Figure 2:**
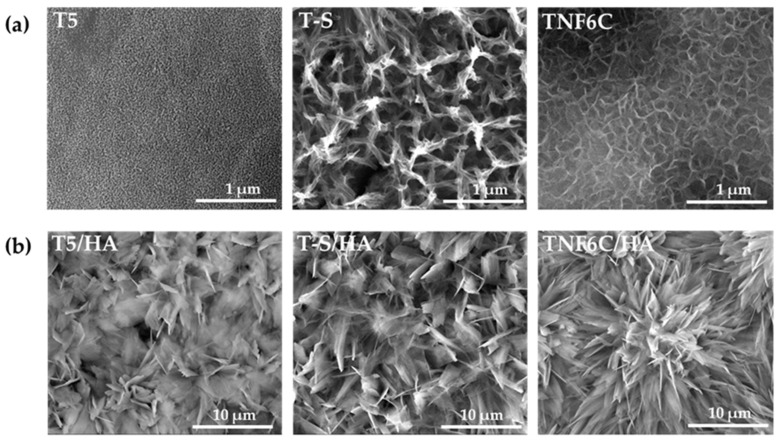
SEM images of the surface morphology of the T5, T-S, TNF6C (**a**), T5/HA, T-S/HA, and TNF6C/HA (**b**) samples.

Considering the results of our earlier investigations, for the bioassays we selected the samples that differed in the morphology of the intermediates (TiO_2_ nanoporous (T5), titanate (T-S), and TiO_2_ nanofibrous (TNF6C)), but that showed excellent physicochemical and mechanical parameters. The SEM images of the chosen intermediate layers, i.e., T5, T-S, and TNF6C, are presented in Figure 2a. Figure 2b shows SEM images of the surface morphology of the samples with the hydroxyapatite layer: T5/HA, TNF6C/HA, and T-S/HA. A floral morphology with numerous hydroxyapatite nanoplatelets was observed for all the double layers.

**Figure 3:**
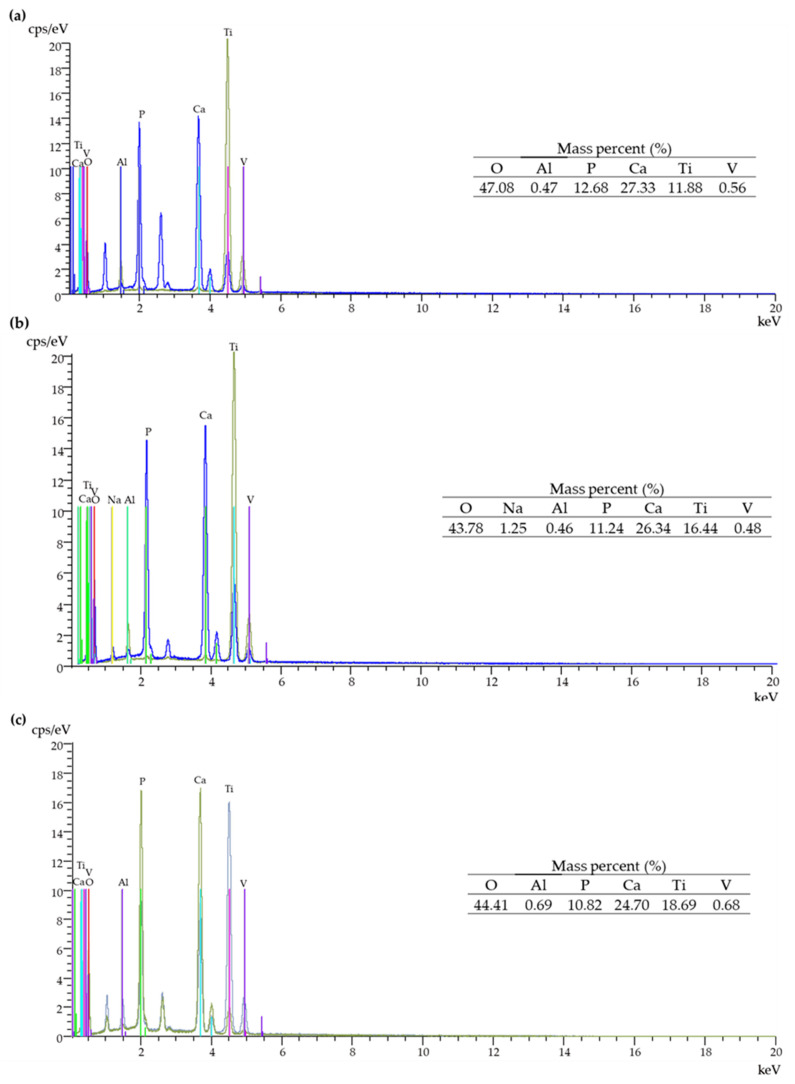
EDS spectra and quantitative data of the T5/HA (**a**), T-S/HA (**b**), and TNF6C/HA (**c**) systems.

EDS spectra of T5/HA, T-S/HA and TNF6C/HA systems are shown in Figure 3a–c. The energy-dispersive X-ray analysis confirmed the presence of Ca and P in the produced systems with a hydroxyapatite layer.

### 3.2. Electrochemical Cathodic Deposition of HA

The electrochemical process of cathodic electrodeposition uses two electrodes immersed in an aqueous solution containing calcium and phosphate ions (in our case: Ca(NO_3_)_2_ · 4 H_2_O and NH_4_H_2_PO_4_). The electrodes are connected to an electrical generator. The nucleation of the hydroxyapatite layer on the surface-modified Ti6Al4V alloy (cathode) can be described by a combination of several reactions (Equations (1)–(18)) [75,76,77,78,79,80].

Water, a solution solvent, is involved in the main redox reactions. The anodic oxidation reaction is:

2H_2_O → O_2_ ↑ + 4H^+^ + 4e^−^ (1)

At the same time, with the use of the electric field, water at the cathode surface is reduced to hydrogen gas and hydroxide ions (Equation (2)). Proton reduction can also occur at the cathode in acidic medium (Equation (3)). The local pH within the diffusion layer is mainly increased by the following two reactions in Equations (2) and (3).

2H_2_O + 2e^−^ → H_2_ ↑ + 2OH^−^ (2)

2H^+^ + 2e^−^ → H_2_↑ (3)

Nevertheless, there are also other cathodic reactions (Equations (4)–(11)) that affect the local increase in the pH value (due to hydroxide generation) of the solution at the cathode–electrolyte interface. Due to the small amounts of O_2_, NO_3_^−^ and H_2_PO_4_^−^ compared to the amount of water, reactions 4–11 are not major.

O_2_ + 2H_2_O + 4e^−^ → 4OH^−^ (4)

O_2_ + 2H_2_O + 2e^−^ → 2OH^−^ + H_2_O_2_ (5)

NO_3_^−^ + 2H^+^ 2e^−^ → NO_2_^−^ + H_2_O (6)

NO_3_^−^ + 10H^+^ + 8e^−^ → NH_4_^+^ + 3H_2_O (7)

NO_3_^−^ + H_2_O + 2e^−^ → NO_2_^−^ + 2OH^−^ (8)

NO_3_^−^ + 7H_2_O + 8e^−^ → NH_4_^+^ + 10OH^−^ (9)

NO_3_^−^ + 6H_2_O + 8e^−^ → NH_3_ + 9OH^−^ (10)

H_2_PO_4_^−^ + H_2_O + 2e^−^ → H_2_PO_3_^−^ + 2OH^−^ (11)

Simultaneously, as the pH changes (between 7.2 and 12.3) in the cathode area, the concentration of hydrogen phosphate ions increases (dissociation of the dihydrogen phosphate ions (Equation (12)). When the pH is equal to or greater than 12.3, phosphate ions predominate (Equation (13)). Local ionic supersaturation occurs, resulting in the precipitation of a calcium phosphate layer.

H_2_PO_4_^−^ → HPO_4_^2−^ + H^+^ (12)

HPO_4_^2−^ → PO_4_^3−^ + H^+^ (13)

Hydroxide groups on the Ti6Al4V surface promote the chemical bonding with calcium and phosphate ions to form the HA layer on the metal surface. Calcium ions may react with hydrogen phosphate ions and phosphate ions to various degrees: e.g., dicalcium phosphate dihydrate (DCPD) (Equation (14)), β-tricalcium phosphate (β-TCP) when T > 800 °C (Equation (15)), octacalcium phosphate (OCP) (Equation (16)), or hydroxyapatite (HA) (Equation (17)).

Ca^2^ +^+^ HPO_4_^2−^ + 2H_2_O → CaHPO_4_·2H_2_O (14)

3Ca^2+^ + 2PO_4_^3−^ → Ca_3_(PO_4_)_2_ (15)

8Ca^2+^ + 2HPO_4_^2−^ + 4PO_4_^3−^ + 5H_2_O → Ca_8_(HPO_4_)_2_(PO_4_)_4_·5H_2_O (16)

10Ca^2+^ + 6PO_4_^3−^ + 2OH^−^ → Ca_10_(PO_4_)_6_(OH)_2_ (17)

The application of alkaline treatment (NaOH) can cause the conversion of other forms of calcium phosphate to HA and an increase in crystallinity (e.g., Equation (18)) [75,76,77,78,79,80].

10CaHPO_4_ + 2OH^−^→ Ca_10_(PO_4_)_6_(OH)_2_ + 4PO_4_^3−^ + 10H^+^ (18)

The higher adhesion of hydroxyapatite (HA) to the TiO_2_ nanocoatings (IL) than to the titanium alloy may be due to the high surface area and physical locking between the HA layer and the intermediate layer [81,82].

### 3.3. Apatite-Forming Ability

**Figure 4:**
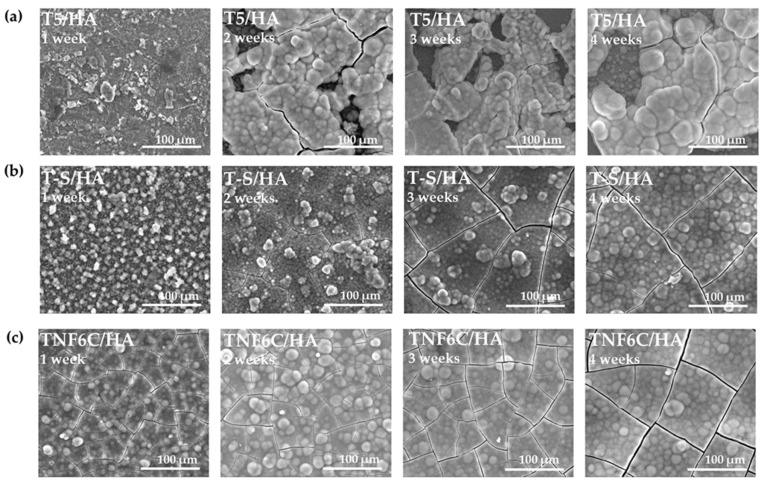
SEM images of the (**a**) T5/HA, (**b**) T-S/HA, and (**c**) TNF6C/HA samples after immersing in SBF for 1–4 weeks.

**Figure 5:**
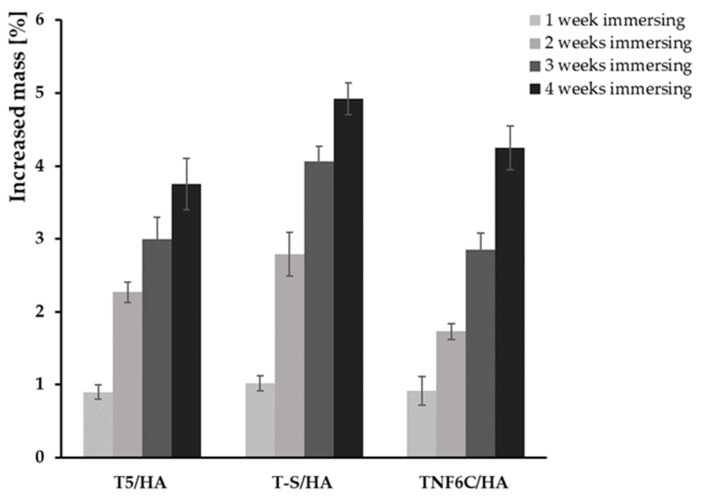
Weight gain for the samples with hydroxyapatite layer after immersing in SBF for 1–4 weeks.

During immersion in the SBF solution, the T5/HA, T-S/HA, and TNF6C/HA systems promoted apatite deposition within a few days. Figure 4 shows the SEM images of the surface morphology changes of specimens after different periods of immersion in SBF (1–4 weeks). Once the samples were removed from the SBF solution and dried, they were weighed and the percentage weight gain was calculated (Figure 5). On the surface of the T5 and TNF6C control specimens, no apatite formation was observed, while apatite formation was reported on the alkali-sodium-modified T-S surfaces, as described in our earlier publication [43]. It was noted that the hydroxyapatite layer produced by the cathodic electrode process, which was deposited on the surface of the intermediates coatings (T5, T-S, and TNF6C) after immersion in the SBF solution, grew at a very fast rate. The thickness of the apatite layer increased with a longer immersion time of the T5/HA, T-S/HA, and TNF6C/HA double layer in SBF solution.

**Figure 6:**
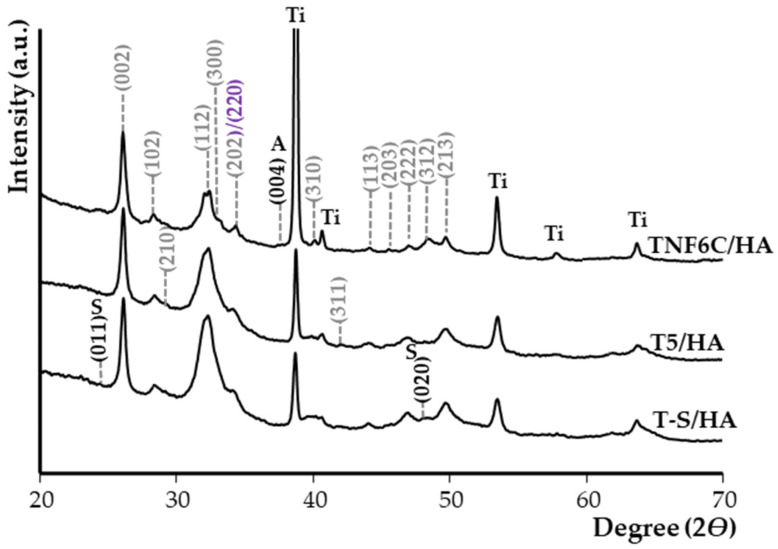
XRD patterns of T-S/HA, T5/HA and TNF6C/HA samples after immersing in SBF for four weeks. (hkl) for CaTiO_3_ are marked in violet. S is assigned to the sodium titanate. Ti is assigned to the Ti6Al4V substrate (TiO_2_ anatase phase (A)).

Figure 6 shows the X-ray diffraction patterns (XRD) of the T5/HA, T-S/HA, and TNF6C/HA samples after immersion in SBF for four weeks. The analysis of these data confirmed that the HA-deposited samples exhibited apatite-forming ability in SBF solution. The positions of the HA peaks marked on the spectra are in accordance with the specifications in JCPDS no. 285 09-0432.

The evaluation of the Ca/P molar ratio of the samples was carried out by EDS analysis (Table 1). During the first week of immersion of the samples in the SBF solution, we noted slight changes in the Ca/P molar ratio compared to the ratio before immersion for the samples T5/HA, T-S/HA, and TNF6C/HA. During the second and third weeks of immersion in SBF solution, the Ca/P ratio was close (for T5/HA, and TNF6C/HA samples) or higher (T-S/HA) than stoichiometric. After four weeks of sample immersion in SBF solution, the Ca/P molar ratios of 1.83 for T-S/HA and TNF6C/HA and 1.87 for T5/HA were detected.

### 3.4. The Viability of Cells Cultured on the Scaffolds

**Figure 7:**
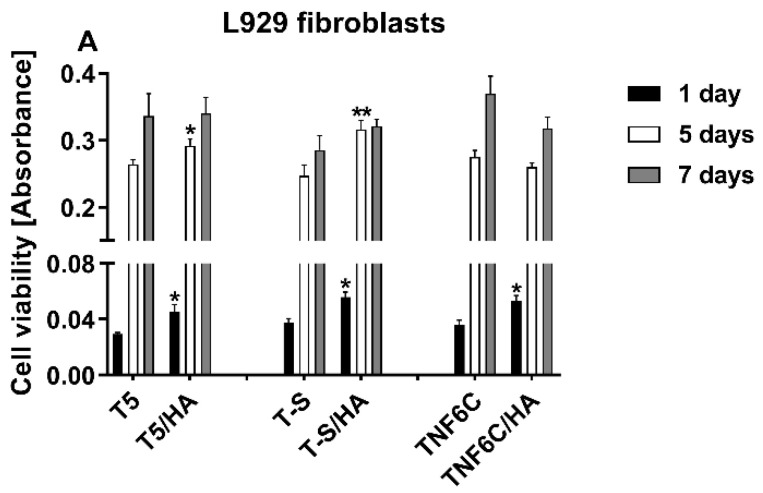

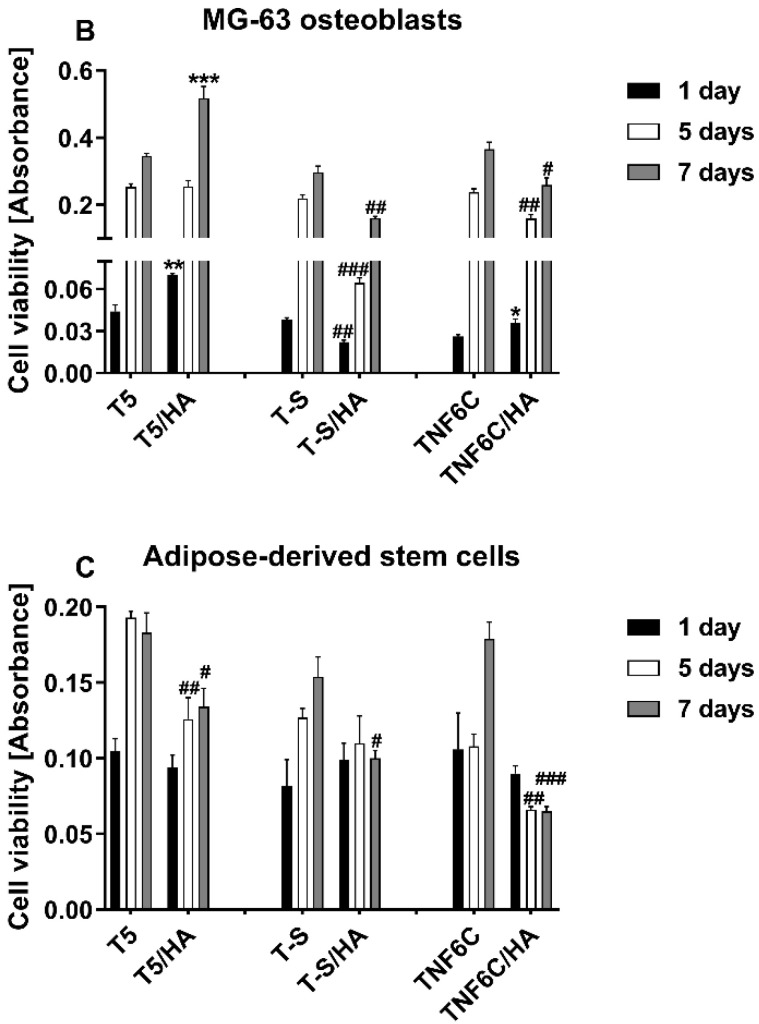
The viability of L929 fibroblasts (**A**), MG-63 osteoblasts (**B**) and adipose-derived stem cells (**C**) cultured on the scaffolds (nanoporous TiO_2_ (T5), titanate (T-S) and nanofibrous TiO_2_ (TNF6C)) coated or not with a hydroxyapatite layer (HA) evaluated using MTT assays after one, five and seven days. The presented data are from four independent experiments. Asterisks and hash marks show statistical differences between the scaffolds coated with HA and the samples without HA at the appropriate time. Asterisks show differences when cell viability measured for the samples with HA was greater compared with the specimens without HA (*** *p* < 0.001, ** *p* < 0.01, * *p* < 0.05). Hash marks denote differences when absorbance values noticed for the scaffolds with HA was lower than the samples not covered with HA (### *p* < 0.001, ## *p* < 0.01, # *p* < 0.05).

The effect of the hydroxyapatite layer (HA) present on the surface of three different nanocoatings (T5/HA, T-S/HA and TNF6C/HA) on L929, MG-63 and ADSC cell viability was assessed after one, five and seven days using the MTT assay. The results were compared with the cell viability estimated for the cells cultured on the specimens without an HA layer (Figure 7). It was observed that with an increase in culture time, a higher or comparable number of both L929 and MG-63 cells grew on the scaffolds with or without HA. This increase in viable cell number was especially observed between one and five days of culture (Figure 7A and Figure 7B, respectively). In the case of ADSCs, the increased cell proliferation rate over time was also noticed for almost all the samples, except for the TNF6C/HA scaffolds, where the number of viable cells after five and seven days was lower compared with one-day incubation. Similarly, on T-S/HA samples the measured values of absorbance did not change over time (Figure 7C). Generally, the nanocoatings with the HA layer induced a higher or comparable level of L929 fibroblast viability in comparison with the samples without HA. A similar effect was also noticed for the MG-63 osteoblasts cultured on the T5 and T5/HA scaffolds. In contrast, the covering of the T-S and TNF6C nanocoatings with HA provoked a decrease in MG-63 cell viability compared with the samples without HA, especially after five and seven days. This effect was also noticed for ADSCs cultivated on the surface of the T5/HA and TNF6C/HA scaffolds.

### 3.5. Cell Proliferation Rate Observed by Scanning Electron Microscopy

**Figure 8:**
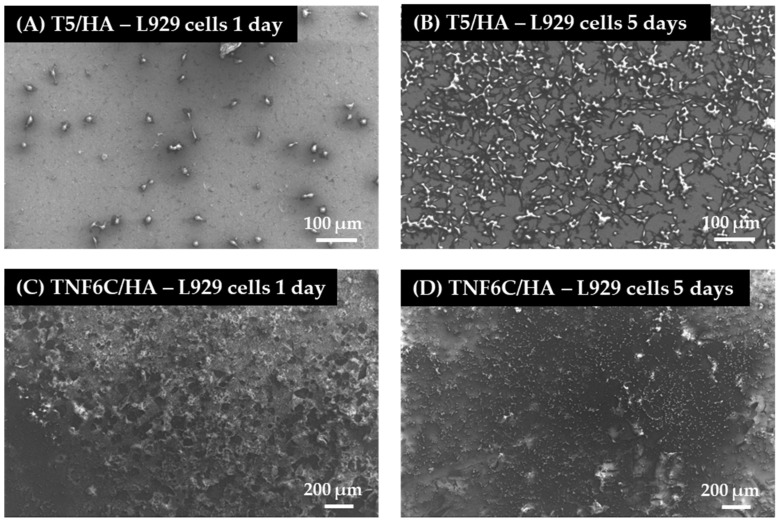
SEM images of L929 fibroblasts growing on the scaffolds coated with hydroxyapatite layer (HA) (nanoporous (T5/HA) and nanofibrous TiO_2_ (TNF6C/HA) for one and five days. The type of specimens and culture time are indicated in the figures.

**Figure 9:**
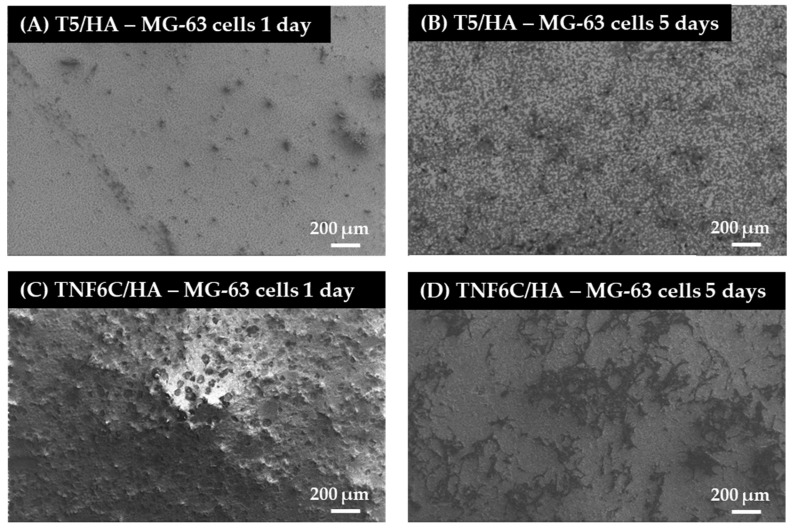
Micrographs from SEM presenting MG-63 osteoblasts cultured on the nanoporous and nanofibrous TiO_2_ scaffolds coated with hydroxyapatite layer (T5/HA and TNF6C/HA, respectively). The type of samples and culture time are described in the figures.

**Figure 10:**
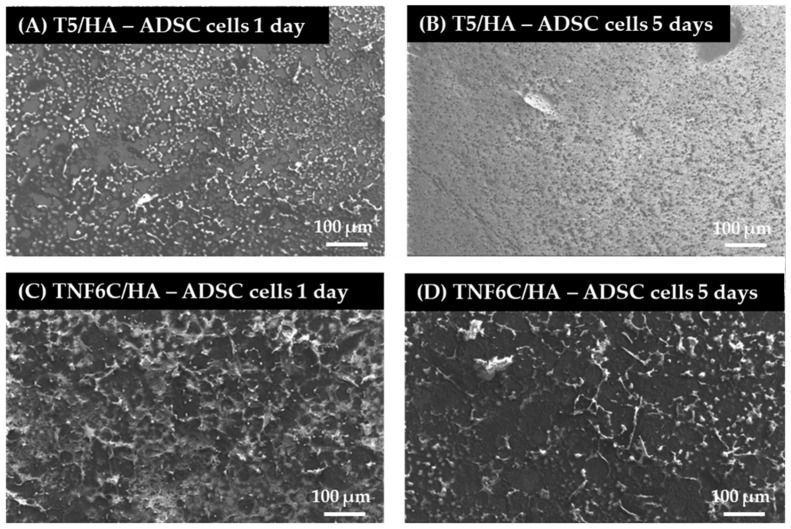
SEM micrographs that present adipose-derived mesenchymal stem cells (ADSCs) growing on the surface of the nanoporous and nanofibrous TiO_2_ specimens coated with hydroxyapatite layer (T5/HA and TNF6C/HA, respectively). The type of specimens and culture time are indicated in the figures.

Scanning electron microscopy (SEM) imaging was harnessed to evaluate the cell morphology and the level of cell proliferation after one and five days. Comparative SEM micrographs of L929 fibroblasts (Figure 8), MG-63 osteoblasts (Figure 9) and ADSCs (Figure 10) were presented for the specimens coated with HA that induced the best and the worst cell viability, taking into consideration all three tested cell lines. These data supported the MTT results and indicated the increase in the cell proliferation level over time observed for L929 cells (Figure 8A,B) and MG-63 cells (Figure 9A,B) growing on the T5/HA specimens. In the case of ADSC cells, many of the cells attached to the surface of the T5/HA samples were already noticed after one day of incubation (Figure 10A). Moreover, the ADSCs grown on these specimens that were integrated with the support produced a huge amount of extracellular matrix that coated almost the entire surface of the samples after five days (Figure 10B).

It must be mentioned that the analysis of SEM micrographs was difficult because of the surface morphology, mainly in the case of the TNF6C/HA scaffolds. Nevertheless, a number of L929 and MG-63 cells grown on these specimens increased over time (Figure 8C,D and Figure 9C,D, respectively). On the contrary, this effect was not noticed for ADSCs (Figure 10C,D).

### 3.6. Osteogenic Potential of Cells Cultured on Different Specimens

**Figure 11:**
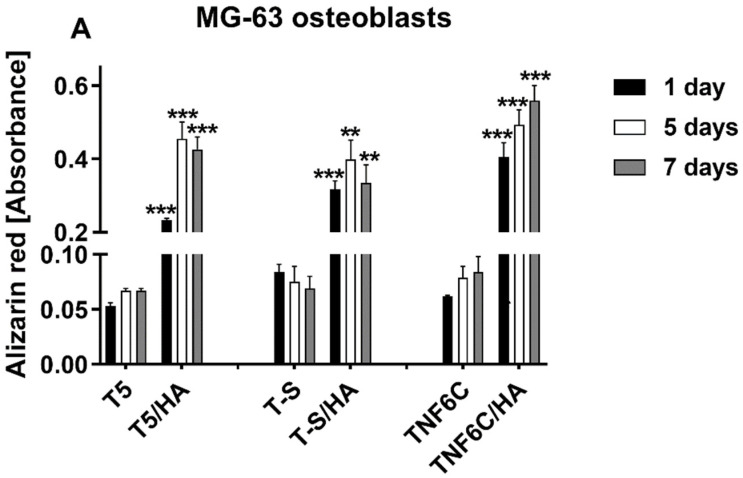

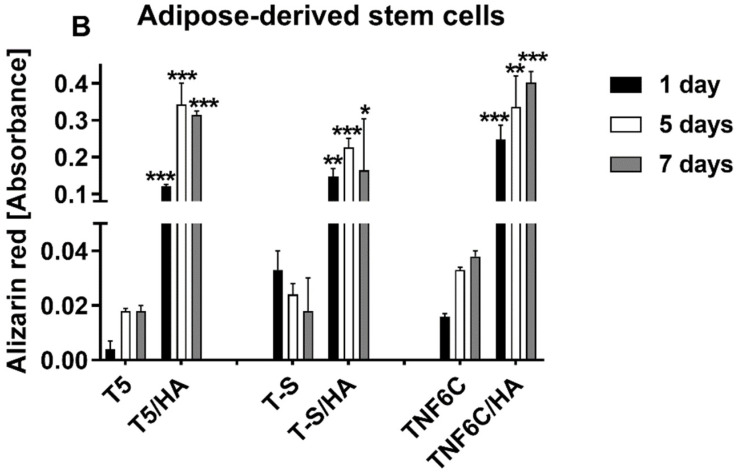
Determination of calcium deposit formation in the extracellular matrix of MG-63 osteoblasts (**A**) and human adipose-derived mesenchymal stem cells (**B**) evaluated after one, five and seven days using Alizarin Red S staining. Asterisks denote differences between Alizarin staining determined for the samples with HA and without HA (*** *p* < 0.001, ** *p* < 0.01; * *p* < 0.05).

**Figure 12:**
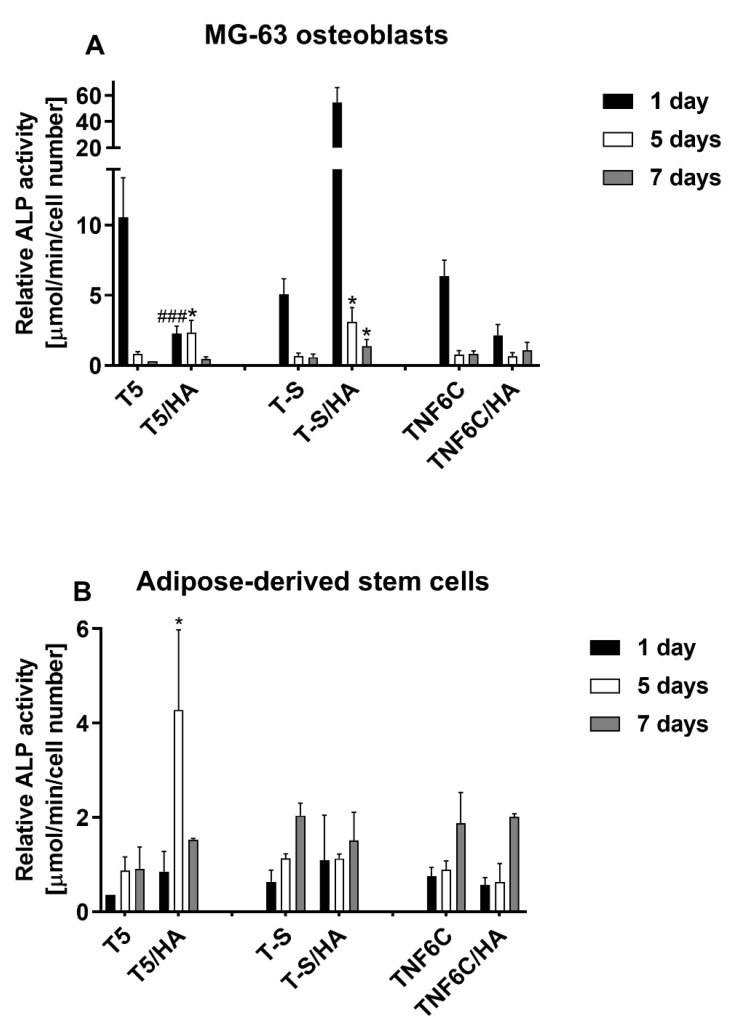
Determination of ALP activity in MG-63 osteoblasts (**A**) and adipose-derived mesenchymal stem cells (**B**) evaluated after one, five and seven days of culture on selected scaffolds. Asterisks and hash marks show statistical differences between the scaffolds with HA and without HA at the appropriate culture time. Asterisks present differences when ALP activity measured for the samples with HA was higher compared with the specimens without HA (* *p* < 0.05). Hash marks indicate differences when ALP activity noticed for the scaffolds with HA was lower than the samples without HA (### *p* < 0.001).

The relatively low cell proliferation rate on the nanofibrous scaffold with a hydroxyapatite coating (TNF6C/HA) could be explained by the osteogenic-differentiation-supportive properties of this specimen. Therefore, we compared the two parameters of effective osteogenesis/calcium deposit formation (Figure 11) and alkaline phosphatase activity (Figure 12).

The extracellular calcium seemed to be deposited in similar quantities in the case of both MG-63 osteoblasts and ADSCs in a time-dependent manner. Additionally, not surprisingly, all the HA-coated specimens were considerably beneficial for the mineralization process.

Some differences in the osteogenic potential of cultured cells can be concluded from the alkaline phosphatase activity determination (Figure 12).

The influence of the hydroxyapatite layer (HA) on endogenous ALP activity in MG-63 cells led to increased activity only in the case of the T-S/HA surface. Nevertheless, the difference between the T-S specimens with and without HA was considerable after 24 h in culture and then decreased. In contrast, human adipose-derived mesenchymal stem cells (ADSCs) exhibited increased ALP activity when grown on the surface of the nanoporous T5/HA layer. These differences indicate that both the chemical nature and nanostructural properties can influence the osteogenic differentiation process.

### 3.7. Antimicrobial Activity

Biocidal activity against the tested strains was not observed for analyzed specimens with HA layers when compared to the untreated microbial inocula (Table 2).

## 4. Discussion

The main direction in which our research tends is the production of a highly biocompatible system with mechanical properties like cortical bone, which can be applied to the design and fabrication of implants. In the course of previous work, we proved that the morphology and structure of interlayers (ILs) have a significant impact on the morphology and mechanical properties of the deposited hydroxyapatite (HA) layers [30]. In this paper, we show that differences in biological activity also depend on the type of Ti6Al4V/IL/HA systems.

The physicochemical properties of the biomaterial surface significantly impact the ability to spontaneously form apatite through the substrate in simulated body fluid (SBF) [43,83]. Our study successfully deposited apatite on the surface of all the Ti6Al4V/IL/HA systems after SBF incubation for 1–4 weeks. The XRD spectra clearly showed the presence of the peaks indicative of hydroxyapatite (HA) constituents. The SEM analysis showed that the HA-layered samples (T5/HA, T-S/HA, TNF6C/HA) were completely covered by the newly formed apatite layers after the first week of immersion in SBF solution. There were a few cracks on the surface of the formed apatite layers, which were most probably due to the release of internal stresses during the drying process [84].

All the chemical and structural properties of the studied materials were reflected in their ability to create a biocompatible surface supporting cell growth. The T5/HA, which is nanoporous, with the highest content of Ca and P in the HA layer, seemed to be most promising in bone tissue regeneration. It is non-toxic and supports cell adhesion and proliferation. This surface allowed for the highest proliferation rates of L929 fibroblasts, MG-63 osteoblasts and mesenchymal stem cells when compared with all the HA-modified samples. The T5/HA also induced the effective deposition of calcium in both osteoblast-like MG-63 cells and adipose-derived mesenchymal stem cells. This process suggests the initiation of osteogenic differentiation in ADSCs and continued osteogenic properties in MG-63. The issue of ALP activity is slightly different; the enzyme activity decreased in MG-63 but increased in ADSCs grown on the T5/HA substrate. These differences reflect the cell properties: MG-63 cells are differentiated cells expressing different markers of osteoblasts [85], while ADSCs are undifferentiated, multipotent cells that require specific extracellular signals to start the differentiation process [86]. As ALP activity is considered the early marker of osteogenic commitment [87], it can be concluded that its increase in ADSCs allows for efficient osteogenesis together with satisfactory cell survival and proliferative potential.

TNF6C/HA with its nanofibrous structure was also biocompatible for L929 and MG-63 cells but decreased the ADSC proliferation. On the other hand, it allowed for the formation of calcium deposits like the other tested specimens, decreased the ALP activity in MG-63 cells, and did not influence the enzymatic activity in ADSCs cells. One can conclude that this surface supports the growth of adjacent cells and stops the proliferation of ADSCs but maintains the osteogenic differentiation efficiency at a similar level to the other HA-modified surfaces. These properties were reported to improve the osseointegration of implanted materials and promote regeneration processes [88].

Developments in the design of bioactive materials, which can provide physical and chemical signals for different cells and regulate their fate, require extensive studies on the relationship between the properties of materials and the fate of cells [89]. Improving both the osseointegration ability and mechanical properties of titanium implants continues to be a challenge in implantology. As a result, there is significant interest in developing technologies that modify the titanium surface. Nevertheless, the implant surface is also susceptible to infection. Infections can be the cause of implant removal or prolonged patient recovery. The important point is that systems with an antimicrobial coating do not impede tissue integration into the implant [90,91,92,93]. In our study, the produced systems with a hydroxyapatite layer (T5/HA, T-S/HA, TNF6C/HA) did not show antimicrobial properties. This result is in line with previously published ones [94,95,96,97] which showed that hydroxyapatite alone or as a layer on titanium specimens did not reveal antimicrobial activity. It in particular showed high biointegration properties [98,99,100,101,102,103]. However, the modifications of Ti/HA layers with antibacterial compounds, e.g., chitosan silver nanoparticles, manganese, strontium or yttrium, may significantly improve such properties [31,94,95,104,105,106,107,108]. Nevertheless, antibacterial coatings are still not well understood in vivo. It should be noted that the antibacterial ability of coatings will gradually weaken over time, and the release of metal ions from their surface may affect the osseointegration efficiency of titanium implants and have a significant impact on their cytotoxicity. So far, the problem of emerging infections is solved with appropriately selected antibiotics [92,93,109].

## 5. Conclusions

Among the previously tested systems, the Ti6Al4V/T5/HA shows the most significant potential for application in the construction of a new generation of implants. The fabricated system (Ti6Al4V/T5/HA) with a nanoporous interlayer, connecting the titanium alloy substrate to the hydroxyapatite layer, shows excellent mechanical properties (adhesion force = 103.11 ± 10.07 mN, hardness = 0.30 ± 0.10 GPa and Young’s Modulus = 35.58 ± 7.41 GPa) [30] and promising bioactivity. It mimics the physiological environment of bone tissue, enhances biointegration, and supports the osteogenic potential of MG-63 cells and ADSCs. Thus, it deserves further investigations focused on acquiring good antimicrobial properties in such a way that the desired balance between the implant’s immune capacity, biointegration, and mechanical properties is still maintained.

## Figures and Tables

**Table 1:** Ca/P ratios obtained from EDS measurements for the samples with hydroxyapatite layer before and after immersing in SBF for 1–4 weeks.

Ca/P (Mole Ratio) of HA Layer					
Sample	Time:	Time:	Time:	Time:	Time:
	0	1 week	2 weeks	3 weeks	4 weeks
T5/HA	1.58	1.54	1.63	1.68	1.87
T-S/HA	1.69	1.56	1.61	1.80	1.83
TNF6C/HA	1.76	1.66	1.57	1.56	1.83

**Table 2:** Antimicrobial activity of selected specimens.

Material	Microorganisms				
	*E. coli* ATCC 8739	*E. coli* ATCC 25922	*S. aureus* ATCC 6538	*S. aureus* ATCC 25923	*C. albicans* ATCC 10231
	Reduction index (R)				
T5/HA	0.34 *	0.38 *	0.04	1.73	0.08
T-S/HA	0.14 *	0.25 *	1.28	1.90	0.25
TNF6C/HA	0.43 *	0.37 *	0.06	0.18 *	0.05

Biocidal activity of specimen is observed when R ≥ 2 (>99%). R = Ut – At, where Ut is the common logarithm of the number of bacteria in the untreated microbial suspension and At is the common logarithm of the number of bacteria in the treated microbial suspension. * no significant increase in microbial growth (<10%).

## Data Availability

Not applicable.
